# Identifying nasopharyngeal carcinoma patients with metachronous metastasis sensitive to local treatment: a real-world study

**DOI:** 10.7555/JBR.36.20220060

**Published:** 2022-05-28

**Authors:** Fanyu Peng, Yizhi Ge, Rongrong Wang, Dingdong Hu, Xiang Cao, Yujie Zhang, Dan Zong, Xia He

**Affiliations:** 1 Department of Radiation Oncology, the Affiliated Cancer Hospital of Nanjing Medical University & Jiangsu Cancer Hospital & Jiangsu Institute of Cancer Research, Nanjing, Jiangsu 210009, China; 2 Xuzhou Medical University, Xuzhou, Jiangsu 221004, China

**Keywords:** nasopharyngeal carcinoma, metastasis, prognostic model, treatment, overall survival

## Abstract

It is difficult for physicians to identify patients with metastatic nasopharyngeal carcinoma (NPC) who are sensitive to local treatment of metastases. Here, we aimed to establish a prognostic model for survival and individualize treatments for patients with metastatic NPC. Data were collated from 240 NPC patients diagnosed with metachronous metastasis between 2006 and 2020 who received palliative chemotherapy with or without local treatment. Multivariable Cox regression was implemented to construct a nomogram which had a concordance index of 0.764 when predicting 1-, 3-, and 5-year overall survival (OS). We then classified patients according to risk, creating low- and high-risk groups using the nomogram. Differences in OS between the two groups were significant (*P<*0.001). In the low-risk group, the OS for patients who received local treatment was longer than those without (*P*=0.009). This novel nomogram shows good performance in classifying patients according to risk and may also be a promising tool for determining who responds best to local treatment. Further validation using external center data is warranted.

## Introduction

Nasopharyngeal carcinoma (NPC) is one of the most common malignancies in head and neck cancers. In 2018 alone, there were approximately 129 000 recorded NPC cases with 73 000 NPC-related deaths worldwide^[1]^. NPC generally responds to radiotherapy and chemotherapy, with radiotherapy being to frontline therapy for NPC. In recent years, the development of intensity-modulated radiotherapy (IMRT) has substantially improved the locoregional control rate for NPC patients. However, distant metastasis constitutes the primary cause of treatment failure^[2]^. Indeed, even after definitive chemoradiotherapy, 15%–30% of non-metastatic NPC cases develop distal metastasis^[3–5]^.

Conventionally, distant metastases are considered an incurable systemic element of disease progression which is generally treated using palliative approaches. The National Comprehensive Cancer Network (NCCN) guidelines recommend platinum-based palliative chemotherapy (PCT) for metastatic NPC. First-line chemotherapeutics studies have highlighted a median survival for metastatic NPC patients ranging from 12.1 to 22.1 months^[6–8]^. However, metastatic disease comprises a broad spectrum of characteristics and prognosis. Hellman and Weichselbaum proposed the concept of "oligometastases", whereby oligometastatic diseases spread to specific organs despite initially having a limited capacity to do so^[9]^. Therefore, localized treatments may be effective within the primary foci or metastatic lesions. In the past few years, local treatments for metastatic NPC have become the focus of interest for many researchers. For example, a randomized phase Ⅲ study found that locoregional radiotherapy targeting primary tumors and metastatic lymph nodes following chemotherapy, significantly improves survival among chemo-sensitive patients with synchronous metastatic NPC^[10]^. Additionally, several retrospective studies have shown that local treatment of metastases can improve survival in patients with metastatic NPC^[11–13]^. Although, existing studies have not identified suitable candidates for local treatment in metastatic NPC.

Lacking an established standard for treating metastatic NPC may be due to the fact that there is no consensus around risk. Metastatic NPC patients are not an homogeneous group of people and there are within and between individual differences in terms of responses to treatment. This creates a whole host of unknown and there are, very few prospective studies which have assessed localized treatments of metastatic lesions. Therefore, we cannot confer survival benefits associated with each treatment modality for patients with metastatic NPC. It is reasonable to suggest that an individualized predictive model is needed to disentangle factors related to survival and it may then be possible to develop more individualized treatment strategies. Here, we developed a prognostic nomogram to identify NPC patients with metachronous metastasis, who would benefit most from local treatments.

## Patients and methods

### Patient population

Data from 347 NPC patients were collated and retrospectively reviewed. All patients had been diagnosed with metastatic NPC at the Affiliated Cancer Hospital of Nanjing Medical University (China) between January 2006 and December 2020. Patients were considered eligible according to the following criteria: (1) patients with histopathologically confirmed primary NPC; (2) those who had received locoregional radiotherapy with IMRT; and (3) those who had metachronous metastatic disease diagnosed more than 6 months after initial diagnosis^[14–15]^.

Patients were excluded if they had: (1) synchronous metastatic disease; (2) or a Karnofsky Performance Score of <70 when diagnosed with distant metastasis; (3) other malignancies; (4) incomplete clinical or survival data; or (5) those who refused antitumor treatment(s). The 8 ^th^ edition of the Union for International Cancer Control/American Joint Committee on Cancer (UICC/AJCC) system was implemented to restage patients. The study was conducted in accordance with the Declaration of Helsinki. The study was approved by the Research Ethics Committee of Jiangsu Cancer Hospital (Approval No. 2021-020), and individual consent for this retrospective study was not deemed necessary.

### Baseline data

During the initial treatment period, all patients received radical IMRT to the nasopharynx and neck using simultaneous integrated boost with 6 MV X-ray radiation in our center. Pretreatment assessments included physical examination, electrocardiogram, chest X-ray, as well as hematologic and biochemical profiling.

An abnormal elevation in Epstein-Barr virus (EBV) DNA was considered a factor signifying potential disease relapse. One hundred and eighteen patients underwent EBV DNA detection pretreatment. Pathological analysis of metastatic lesions was established as the gold standard for diagnosing metastases. When pathological findings were unavailable, diagnosis was based on a multimodality imaging system, which included contrast-enhanced computed tomography (CT) of the chest and abdomen, whole-body bone scans, and positron emission tomography (PET-CT).

The total of metastatic lesions was calculated according to image findings, of which 1–5 lesions were defined as oligometastic. More than five lesions was defined as polymetastatic. Locoregional recurrence was confirmed using fiberoptic endoscopy of the nasopharynx and magnetic resonance imaging (MRI) scans of the head and neck.

### Treatment

All patients received PCT according to previous chemotherapy regimens and toxicity tolerance after first distant failure. The median PCT cycles was four, ranging from 1 to 12. PCT regimens included platinum plus 5-fluorouracil, taxane plus platinum, taxane plus platinum with 5-fluorouracil, and gemcitabine plus platinum (GP).

After receiving chemotherapy, 146 patients received local treatment for metastatic lesions, while the remaining patients did not. One or more localized treatments were administered to these patients of whom 16 underwent surgery (1, 11, and 4 cases with liver, lung, and distant lymphatic metastasis, respectively). One hundred and thirty-one patients received radiotherapy for one or more metastatic lesions of whom five received radiofrequency ablation for liver metastases.

Among the patients who received radiotherapy, 19 received stereotactic body radiosurgery (SBRT), including seven with liver metastases and 12 with lung metastases. Radiation doses ranged from 30 to 66 Gy (2–10 Gy/fraction), with a median biologically effective dose of 60 Gy (range: 35–100).

### Follow-up

Follow-ups were arranged every three months for the first 2 years and then every 6 to 12 months thereafter, until death or the last follow-up date (June 30, 2021). During the follow-up period, nasopharyngoscopy, contrast-enhanced MRI of nasopharynx and neck, and contrast-enhanced CT of chest and abdomen were performed. PET-CT was considered if necessary. The disease-free interval (DFI) was calculated using the date of initial NPC diagnosis until the diagnosis of distant metastasis. OS was considered, the primary endpoint of this study and was calculated from the date of diagnosis of metastasis to the date of last follow-up or death.

### Statistical analysis

SPSS version 22.0 (IBM, USA) was used to perform statistical analysis. All variables are categorical, and intergroup comparisons were performed using Pearson's chi-squared test or Fisher's test. Cox's proportional hazard model was implemented to investigate the independence of prognostic factors related to OS. Hazard ratios (HR) and corresponding 95% confidence intervals (CI) are reported as effect estimates. Variables that met the predetermined significance threshold (*P<*0.1) under univariable analysis were entered into multivariable analysis. Two-tailed *P<*0.05 were considered statistically significant.

R software (version 4.0.3) was used for model building. The prognostic nomogram was constructed using the "rms" package with independent prognostic factors^[16]^. The "nomogramFormula" package calculated scores for each variable as well as a total score for each patient^[17]^. The "nomogramEX" package was used to extract formulas from the nomogram^[18]^. The concordance index (C-index) and calibration curves were used to assess model performance. Kaplan-Meier curves were used to estimate the 1-, 3-, and 5-year OS rates and differences between groups of patients.

## Results

### Clinical characteristics

**Figure 1:**
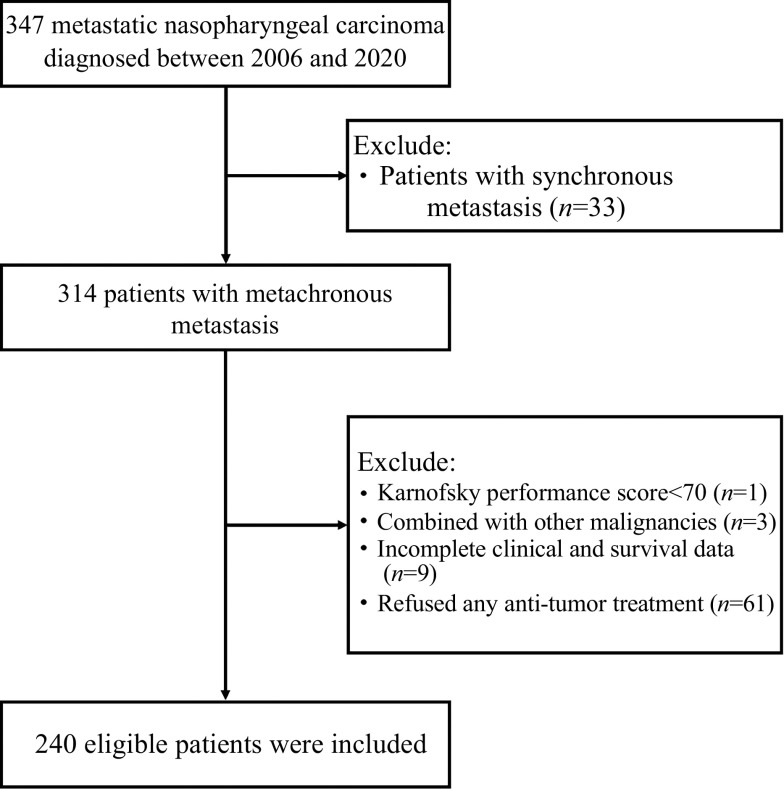
Flowchart for patient selection.

A total of 240 NPC patients with metachronous metastasis were considered eligible (***Fig. 1***). All patients had histologically confirmed non-keratinizing NPC at initial diagnosis. The median age was 49 years (range: 11–78), and 78.8% of this sample were male. 32.5% (*n*=78) of this sample developed distant metastasis within 12 months following the primary diagnosis (DFI≤12 months). The remaining patients developed distant metastasis after 12 months (DFI>12 months).

The incidence of lung, bone, liver, and distant nodal metastasis was 42.5%, 47.5%, 28.3%, and 28.3%, respectively. Oligometastatic disease was detected in 60.4% (*n*=145) patients (63 with single metastatic lesions and 82 with 2 to 5 lesions). 39.6% (*n*=95) patients had polymetastatic disease (>5 lesions). 13.8% (*n*=33) had simultaneous locoregional recurrence. One hundred and twenty (50%) patients received ≥4 cycles of PCT after the first distant failure, and the remainder received <4 cycles. Demographics and the clinical characteristics of patients with metastatic NPC have been summarized and are provided in ***Table 1***.

**Table 1:** Baseline characteristics of patients with metastatic nasopharyngeal carcinoma

(*n* [%])				
Characteristics	Total patients (*n*=240)	LT+PCT (*n*=146)	PCT (*n*=94)	*P-* value
Age (years) ^a^				0.202
<49	117 (48.8)	76 (52.1)	41 (43.6)	
≥49	123 (51.2)	70 (47.9)	53 (56.4)	
Sex				0.104
Female	51 (21.2)	26 (17.8)	25 (26.6)	
Male	189 (78.8)	120 (82.2)	69 (73.4)	
BMI				0.653
<23	109 (45.4)	68 (46.6)	41 (43.6)	
≥23	131 (54.6)	78 (53.4)	53 (56.4)	
KPS				0.644
≤80	104 (43.3)	65 (44.5)	39 (41.5)	
>80	136 (56.7)	81 (55.5)	55 (58.5)	
T stage^b^				0.126
T1	27 (11.3)	11 (7.5)	16 (17.0)	
T2	43 (17.9)	27 (18.5)	16 (17.0)	
T3	75 (31.2)	50 (34.2)	25 (26.6)	
T4	95 (39.6)	58 (39.8)	37 (39.4)	
N stage^b^				0.940
N0	3 (1.2)	2 (1.4)	1 (1.1)	
N1	77 (32.1)	48 (32.9)	29 (30.9)	
N2	109 (45.4)	64 (43.8)	45 (47.9)	
N3	51 (21.3)	32 (21.9)	19 (20.1)	
DFI (months)				0.682
≤12	78 (32.5)	46 (31.5)	32 (34.0)	
>12	162 (67.5)	100 (68.5)	62 (66.0)	
Lung metastasis				<0.001
No	138 (57.5)	98 (67.1)	40 (42.6)	
Yes	102 (42.5)	48 (32.9)	54 (57.4)	
Bone metastasis				<0.001
No	126 (52.5)	61 (41.8)	65 (69.1)	
Yes	114 (47.5)	85 (58.2)	29 (30.9)	
Liver metastasis				0.001
No	172 (71.7)	116 (79.5)	56 (59.6)	
Yes	68 (28.3)	30 (20.5)	38 (40.4)	
Distant nodal metastasis				<0.001
No	165 (68.8)	115 (78.8)	50 (53.2)	
Yes	75 (31.2)	31 (21.2)	44 (46.8)	
No. of metastatic lesions				<0.001
1	63 (26.2)	50 (34.3)	13 (13.8)	
2–5	82 (34.2)	50 (34.2)	32 (34.0)	
>5	95 (39.6)	46 (31.5)	49 (52.2)	
Pretreatment EBV DNA				0.148
Undetectable	49 (20.4)	35 (24.0)	14 (14.9)	
Detectable	69 (28.8)	37 (25.3)	32 (34.0)	
Not available	122 (50.8)	74 (50.7)	48 (51.1)	
Locoregional recurrence				0.051
No	207 (86.2)	131 (89.7)	76 (80.9)	
Yes	33 (13.8)	15 (10.3)	18 (19.1)	
No. of PCT cycles				0.002
<4	120 (50.0)	85 (58.2)	35 (37.2)	
≥4	120 (50.0)	61 (41.8)	59 (62.8)	
^a^Age at diagnosis of distant metastasis. ^b^According to the 8^th^ UICC/AJCC staging system. LT: local treatment of metastases; PCT: palliative chemotherapy; BMI: body mass index; KPS: Karnofsky performance score; DFI: disease-free interval; EBV: Epstein-Barr virus; No.: number.				

The median follow-up duration was 23 months (ranging from 1 to 176 months). 169 patients died during this study period. The 1-, 3-, and 5-year OS rates were 76.1%, 39.8%, and 24.8%, respectively.

### Independent prognostic factors

The results of univariable and multivariable regression analysis are provided in ***Table 2***. Six variables were considered statistically significant intergroup differences, including sex, DFI, liver metastasis, number of metastatic lesions, locoregional recurrence, and number of PCT cycles. These variables were identified as independent prognostic factors for OS in metastatic NPC patients. Specifically, being female was associated with a better prognosis (HR=1.690; 95% CI: 1.095–2.542; *P*=0.012).

**Table 2:** Univariable and multivariable analysis of overall survival in patients with metastatic nasopharyngeal carcinoma

Variables	Univariable			Multivariable	
	HR (95% CI)	*P-*value		HR (95% CI)	*P-*value
Age (years)^a^					
<49	Reference				
≥49	1.137 (0.839–1.542)	0.407			
Sex					
Female	Reference			Reference	
Male	1.484 (1.009–2.182)	0.045		1.690 (1.124–2.542)	0.012
BMI					
<23	Reference				
≥23	0.991 (0.732–1.342)	0.952			
KPS					
≤80	Reference				
>80	0.918 (0.677–1.244)	0.580			
T stage^b^					
T1	Reference				
T2	1.057 (0.602–1.857)	0.847			
T3	0.847 (0.507–1.415)	0.526			
T4	0.722 (0.436–1.196)	0.206			
N stage^b^					
N0	Reference				
N1	3.265 (0.451–23.624)	0.241			
N2	3.572 (0.496–25.717)	0.206			
N3	4.840 (0.663–35.327)	0.120			
DFI (months)					
≤12	Reference			Reference	
>12	0.604 (0.442–0.825)	0.001		0.650 (0.466–0.908)	0.012
Lung metastasis					
No	Reference			Reference	
Yes	0.754 (0.554–1.025)	0.072		0.848 (0.580–1.239)	0.394
Bone metastasis					
No	Reference			Reference	
Yes	1.397 (1.033–1.890)	0.030		0.895 (0.612–1.310)	0.569
Liver metastasis					
No	Reference			Reference	
Yes	1.737 (1.261–2.393)	0.001		1.580 (1.113–2.243)	0.011
Distant nodal metastasis					
No	Reference			Reference	
Yes	1.659 (1.210–2.276)	0.002		0.866 (0.579–1.295)	0.483
No. of metastatic lesions					
1	Reference			Reference	
2–5	1.746 (1.115–2.736)	0.015		2.875 (1.749–4.725)	<0.001
>5	4.713 (3.052–7.279)	<0.001		7.955 (4.597–13.766)	<0.001
Locoregional recurrence					
No	Reference			Reference	
Yes	1.473 (0.974–2.229)	0.067		2.079 (1.338–3.230)	0.001
No. of PCT cycles					
<4	Reference			Reference	
≥4	0.630 (0.465–0.853)	0.003		0.403 (0.281–0.576)	<0.001
^a^Age at diagnosis of distant metastasis. ^b^According to the 8^th^ UICC/AJCC staging system. BMI: body mass index; KPS: Karnofsky performance score; DFI: disease-free interval; No.: number; PCT: palliative chemotherapy; HR: hazard ratio; CI: confidence interval.					

Patients who developed distant metastasis within 12 months after the primary diagnosis (DFI≤12) generally had poorer prognosis (HR=0.650; 95% CI: 0.466–0.908; *P*=0.012). Concerning the site of metastasis, liver metastasis (HR=1.580; 95% CI: 1.113–2.243; *P=*0.011) was an adverse prognostic factor for survival. The number of metastatic lesions had a profound impact on clinical outcomes compared to those with single lesions. Patients with 2 to 5 or more than 5 lesions had a significantly poorer prognosis (HR=2.875; 95% CI: 1.749–4.725 and HR=7.955; 95% CI: 4.597–13.766; both *P<*0.001).

Concurrent locoregional recurrence with distant metastasis conferred an increased mortality risk (HR=2.079; 95% CI: 1.338–3.230; *P*=0.001). Additionally, patients who received ≥4 cycles of PCT experienced significantly improved OS than compared to those with <4 cycles (HR=0.403; 95% CI: 0.281–0.576; *P<*0.001).

### Developing and assessing the nomogram

**Figure 2:**
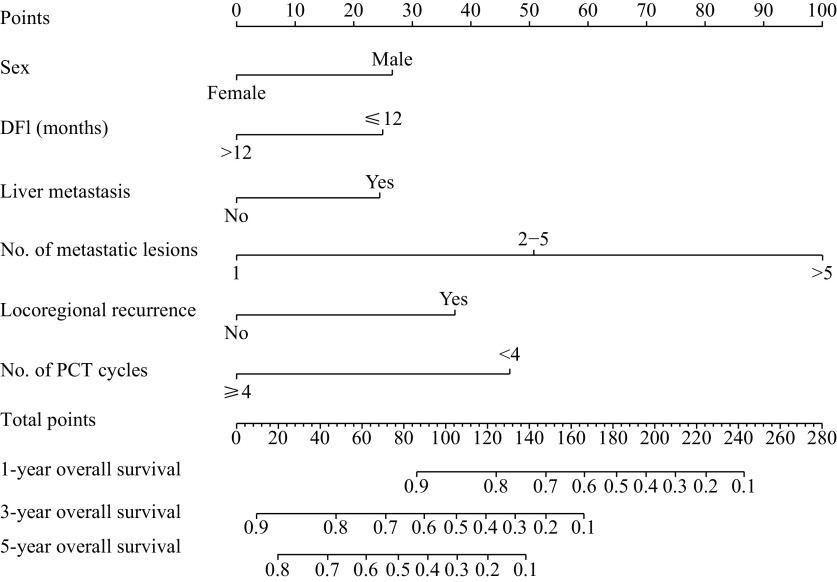
Prognostic nomogram for predicting the 1-, 3-, and 5-year overall survival in patients with metastatic nasopharyngeal carcinoma.

Based on the predictors derived through multivariable regression analysis, a prognostic nomogram was constructed to predict the 1-, 3-, and 5-year survival (***Fig. 2***). In order to use the nomogram, each variable subtype had to correspond with score on a specific point scale. The total score was then calculated using scores which corresponded to each variable, to estimate the 1-, 3-, and 5-year OS rates (***Supplementary Table 1***, available online). Nomogram formulas for generating OS probabilities were calculated, as follows:

1-year OS=1.54e−07×points^3−9.34e−05×points^2+0.012 174 368×points+0.443 082 846

3-year OS=1.54e−07×points^3−5.7954e−05× points^2+0.000 544 564×points+0.896 856 023

5-year OS=2.81e−07×points^3−7.6778e−05× points^2−1.627 6e−05×points+0.825 548 036

**Figure 3:**
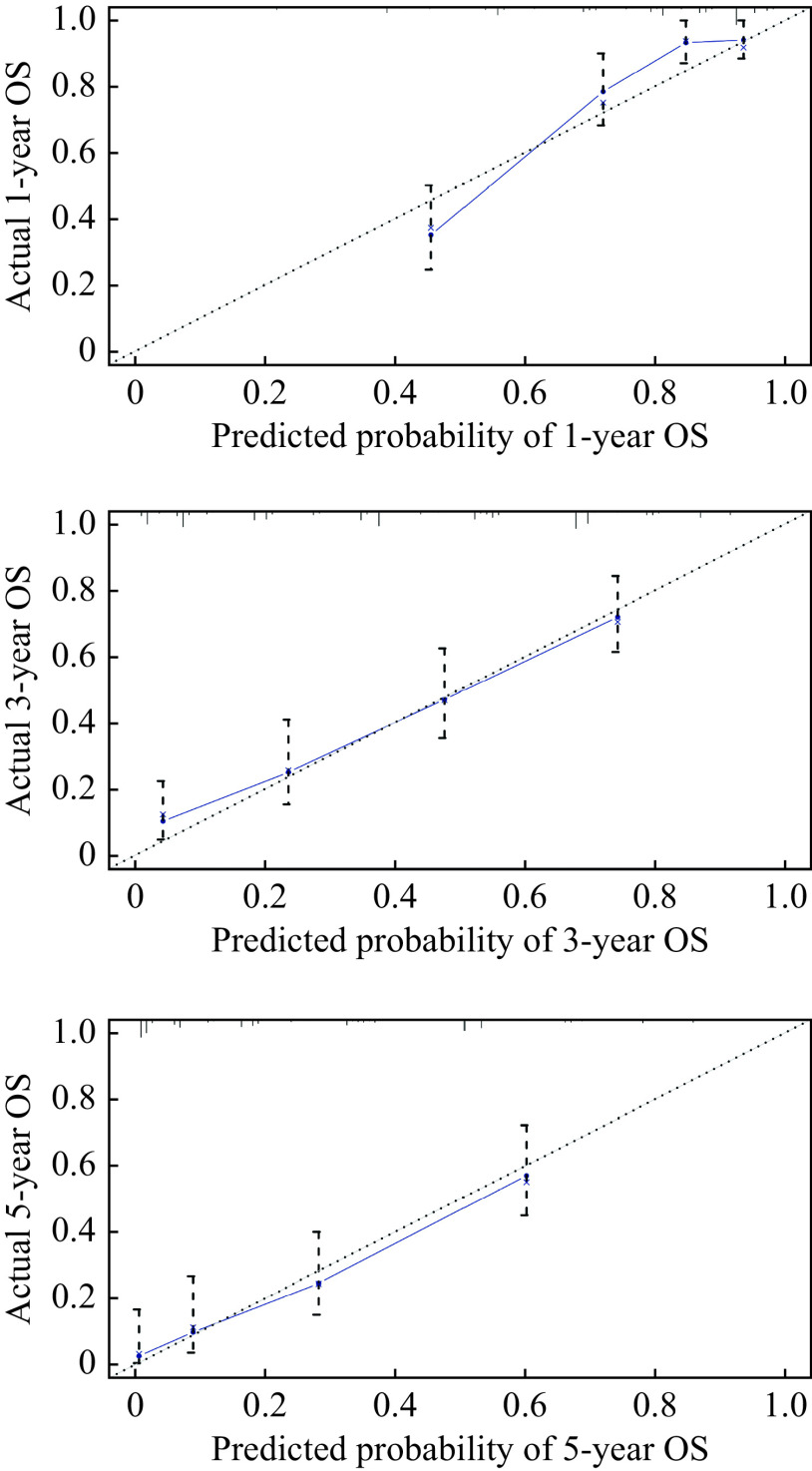
Calibration curves of the nomogram for predicting the 1-, 3-, and 5-year overall survival.

The C-index of the nomogram was 0.764 (95% CI: 0.649–0.806), which suggests good accuracy for predicting survival. Calibration curves obtained using the bootstrap method showed good fitness between predicted and observed survival (***Fig. 3***).

### Risk stratification

We used the median total score from the prognostic nomogram to stratify the cohort into the low- (risk score 0–122) and high-risk (risk score 123–260) groups (***Table 3***). Baseline characteristics of low- and high-risk patients have been provided in the ***Supplementary Tables 2*** and ***3*** (available online), respectively. The 1-, 3-, and 5-year OS rates of patients in each group are also summarized in ***Supplementary Table 4*** (available online).

**Table 3:** Characteristics of low- and high-risk groups defined by the prognostic nomogram　　　　　　　 *n* (%)

Variable	Low-risk (*n*=116)	High-risk (*n*=124)	*P-*value
Age (years)^a^			0.240
<49	52 (44.8)	65 (52.4)	
≥49	64 (55.2)	59 (47.6)	
Sex			0.020
Female	32 (27.6)	19 (15.3)	
Male	84 (72.4)	105 (84.7)	
BMI			0.390
<23	56 (48.3)	53 (42.7)	
≥23	60 (51.7)	71 (57.3)	
KPS			0.330
≤80	54 (46.6)	50 (40.3)	
>80	62 (53.4)	74 (59.7)	
T stage^b^			0.852
T1	11 (9.5)	16 (1.9)	
T2	21 (18.1)	22 (17.7)	
T3	38 (32.8)	37 (29.8)	
T4	46 (39.6)	49 (39.6)	
N stage^b^			0.530
N0	2 (1.7)	1 (0.8)	
N1	41 (35.3)	36 (29.0)	
N2	52 (44.8)	57 (46.0)	
N3	21 (18.2)	30 (24.2)	
DFI (months)			0.034
≤12	30 (25.9)	48 (38.7)	
>12	86 (74.1)	76 (61.3)	
Lung metastasis			0.219
No	62 (53.4)	76 (61.3)	
Yes	54 (46.6)	48 (38.7)	
Bone metastasis			0.009
No	71 (61.2)	55 (44.4)	
Yes	45 (38.8)	69 (55.6)	
Liver metastasis			0.005
No	93 (80.2)	79 (63.7)	
Yes	23 (19.8)	45 (36.3)	
Distant nodal metastasis			<0.001
No	96 (82.8)	69 (55.6)	
Yes	20 (17.2)	55 (44.4)	
No. of metastatic lesions			<0.001
1	60 (51.7)	3 (2.4)	
2–5	53 (45.7)	29 (23.4)	
>5	3 (2.6)	92 (74.2)	
Locoregional recurrence			0.138
No	104 (89.7)	103 (83.1)	
Yes	12 (10.3)	21 (16.9)	
No. of PCT cycles			<0.001
<4	44 (37.9)	76 (61.3)	
≥4	72 (62.1)	48 (38.7)	
^a^Age at diagnosis of distant metastasis. ^b^According to the 8^th^ UICC/AJCC staging system. BMI: body mass index; KPS: Karnofsky performance score; DFI: disease-free interval; No.: number; PCT: palliative chemotherapy; LT: local treatment of metastases.			

**Figure 4:**
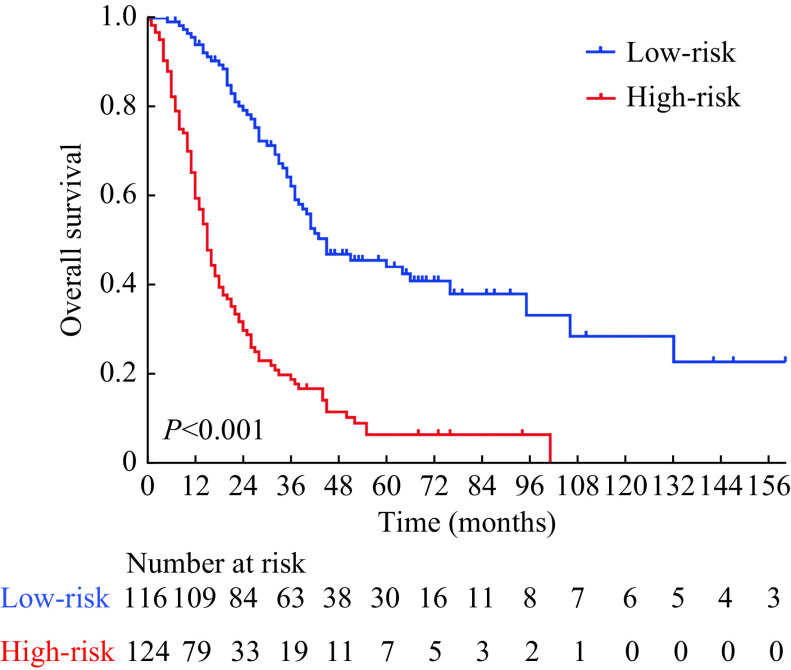
Kaplan-Meier survival curves of patients with metastatic nasopharyngeal carcinoma in different risk groups.

Kaplan-Meier curves for OS highlighted significant intergroup differences (*P<*0.001; ***Fig. 4***), which also suggests this model is effective at stratifying metastatic NPC patients. With an estimated median OS of 45.0 months, the 1-, 3-, and 5-year OS rates of patients in the low-risk group were 93.6%, 60.0%, and 41.1% respectively, which were all significantly higher than the OS rates observed in high-risk patients (median OS: 15.0 months; 1-, 3-, and 5-year OS rates: 59.4%, 18.8%, and 6.4%). The nomogram remained a clinically and statistically significant prognostic model when this sample was stratified according to age (≥49 or <49 years), sex (female or male), T stage (T1–2 or T3–4), and N stage (N0–1 or N2–3) ( ***Supplementary Fig. 1***, available online).

### Local treatment in different risk groups

**Figure 5:**
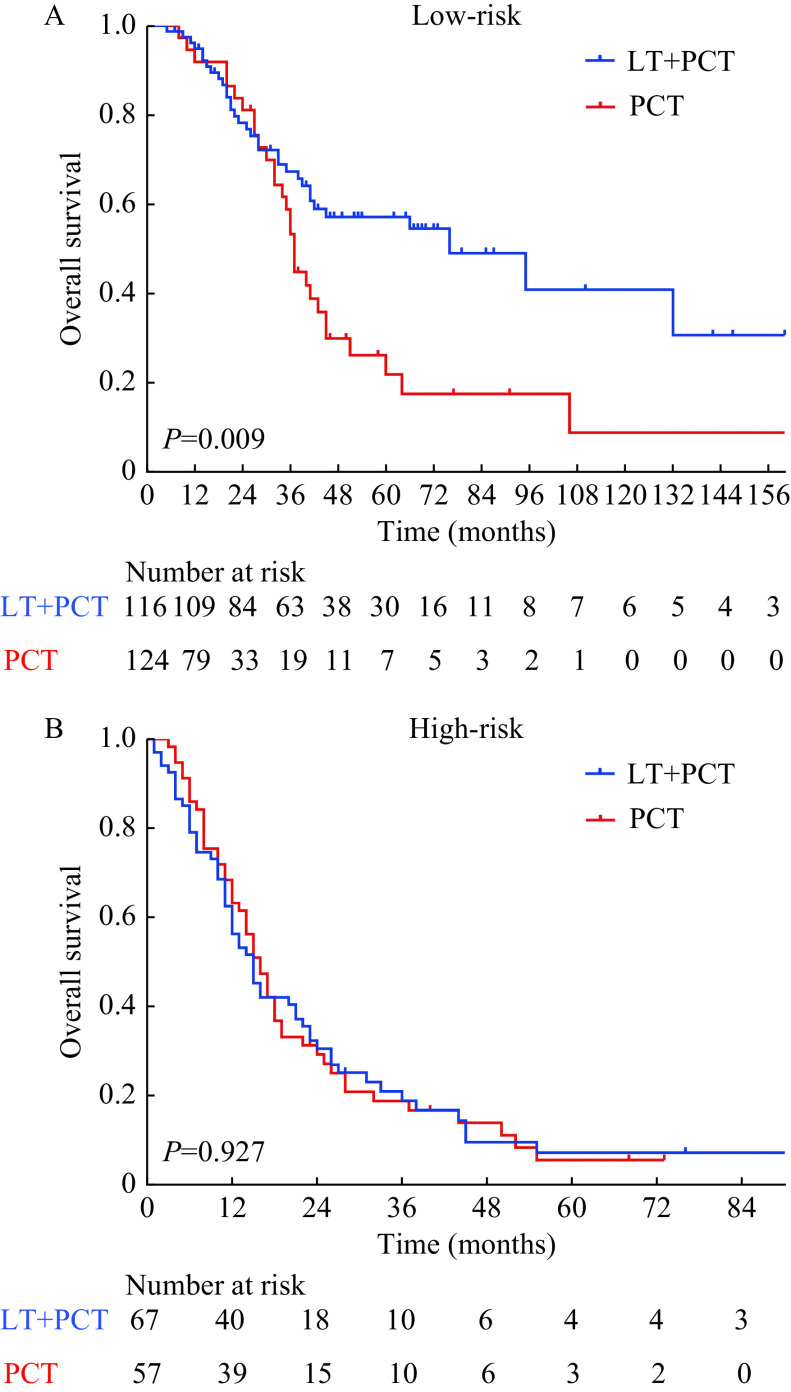
Kaplan-Meier survival curves of patients with metastatic nasopharyngeal carcinoma receiving different treatments.

To select patients who might benefit from local treatment of metastases, we further compared OS for patients with or without local treatment and within each risk group. For low-risk patients who received PCT plus local treatment or PCT alone, the 1-, 3-, and 5-year OS rates were 94.9%, 67.3%, 57.0%, and 91.9%, 53.1%, 21.7%, respectively (*P*=0.009, ***Fig. 5A***). In the high-risk group, the 1-, 3-, and 5-year OS rates of patients with or without local treatment were 56.2%, 18.9%, 7.2%, and 63.2%, 18.8%, 5.6%, respectively (*P*=0.927, ***Fig. 5B***).

According to multivariable analysis (***Table 4***), patients in the low-risk group, who received PCT plus local treatment, had a significantly better prognosis than those who received PCT alone (HR=0.570; 95% CI: 0.343–0.947; *P*=0.030). However, there was no significant intergroup difference in terms of survival between the two treatment groups in the high-risk group (HR=0.994; 95% CI: 0.678–1.458; *P*=0.975).

**Table 4:** Multivariable analysis of overall survival for low- and high-risk groups

Variable	Low-risk			High-risk	
	HR (95% CI)	*P-*value		HR (95% CI)	*P-*value
Treatment (LT+PCT *vs.* PCT)	0.570 (0.343–0.947)	0.030		0.994 (0.678–1.458)	0.975
Risk score	1.018 (1.007–1.028)	0.001		1.022 (1.014–1.029)	<0.001
LT: local treatment of metastases; PCT: palliative chemotherapy; HR: hazard ratio; CI: confidence interval.					

## Discussion

Significant progress has been made in localized controls for NPC due to the development of IMRT and imaging systems which are now more precise at defining tumors and identifying organs at risk. However, distant metastases are still a challenge for patients with NPC, and there is no consensus regarding treatment strategies. According to the GEM20110714 study^[7]^, the preferred first-line regimen of GP in metastatic NPC resulted in a median OS of 22.1 months and 1-, 3-, and 5-year OS rates of 79.9%, 31.8%, and 19.2%, respectively. However, prognosis for metastatic NPC patients has significant heterogeneity in the real world due to the broad spectrum of metastatic diseases and the use of various treatments. In this study, we established an easy-to-use nomogram for predicting OS for patients with metastatic NPC. This novel model appears to perform well at identifying candidates who would benefit from local treatments.

According to the NCCN guidelines, systemic chemotherapy remains the cornerstone of the therapeutic management of metastatic NPC patients. Consistent with previous reports^[19–20]^, this study showed that patients who received more than 4 cycles of PCT had a significantly longer overall survival compared to those administered with fewer than four cycles. However, adequate chemotherapy does not mean that the course of chemotherapy should be extended indefinitely. Numerous reports have proposed that appropriate local treatment could improve the local control of metastatic lesions and confers survival benefits on some patients. For example Huang *et al*^[11]^ reported that NPC patients with limited liver metastases treated by a partial hepatectomy achieved a median OS of 45.2 months, which was obviously elevated compared to 14.1 months in the control group. Likewise in patients with lung-only metastases from NPC, pulmonary metastasectomy provided reliable local control and survival benefits with significantly higher 5-year survival rates than in the nonsurgically treated group (75.5% *vs.* 47.8%, *P*=0.005)^[21]^. A retrospective studies also revealed that chemoradiotherapy conferred a better prognosis than chemotherapy alone in NPC patients with bone metastases^[22–23]^.

In clinical practice, however, not all metastatic lesions of NPC will respond to local treatments. Based on the nomogram we established in this study, patients with metastatic NPC were stratified into low- and high-risk groups. We found that the survival benefit associated with local treatment which was solely observed in those considered at low-risk. There was no additional survival benefit in high-risk patients receiving local treatment compared with PCT alone. This may explain why local treatment did not demonstrate a statistically significant difference in OS across the entire cohort and further demonstrates the validity of our model. Therefore, clinicians can use our model to determine who responds best to local treatment and make individualized treatment recommendations. Our study fills the gap where previous studies failed to identify who would benefit from local treatment of metastases. Increasingly, evidence suggests that applying, potentially curative metastasis-directed radiotherapy for oligometastatic disease in various solid tumors, significantly improves survival and was associated with low treatment-related toxicity^[24–27]^. Due to the advancement of radiotherapeutic technologies, such as SBRT, ablative radiotherapy doses can be safely delivered to sites of metastasis. Regrettably, only 19 patients in our study underwent SBRT for metastatic lesions, and the sample size was too small for further analysis.

The definition of oligometastatic NPC has been widely discussed, and the generally accepted definition notes the presence of up to five metastatic lesions on imaging. In the present study, multivariable Cox regression indicated that the number of metastatic lesions was the most decisive prognostic parameter. Consistent with results reported in previous studies^[20,28–29]^, the prognosis of patients with oligometastatic disease (1-5 lesions) is significantly better than that for those with polymetastatic disease (>5 lesions). The presence of a single lesion predicted superior survival outcomes than that of multiple metastatic lesions, possibly because a single lesion is more amenable to radical local treatment in the clinic. It is worth noting that we only included patients with metachronous metastatic disease or what is commonly referred to as post-treatment metastasis. Unlike those with synchronous metastatic NPC, patients with metachronous metastasis have already received aggressive radiotherapy to the primary foci. In the present study, concurrent locoregional recurrence was identified as a poor prognostic factor for metastatic NPC. Locoregional recurrent NPC after initial radiotherapy is likely to be radioresistant. Besides, an uncontrolled primary tumor may promote distant metastasis. It is currently unclear whether a locoregionally controlled primary tumor should become a precondition for diagnosing the oligometastatic disease, but it should be considered a crucial prognostic parameter for metastatic NPC.

Generally, patients with oligometastatic disease tend to have a low tumor burden and are more likely to receive aggressive metastasis-directed therapy, such as surgery or high-dose irradiation. In contrast, patients with polymetastatic disease are generally considered to have no curative potential and receive palliative systemic therapy. Nonetheless, in our study, not all cases with oligometastases were included in the low-risk group, and a proportion of patients with polymetastatic disease achieved better survival following comprehensive treatment. Metastatic status changes throughout the disease dynamically, and patients with polymetastatic disease may achieve oligometastatic status during successful systemic therapy^[14–15]^. Moreover, oligometastatic disease is diagnosed solely based on imaging because no biomarker is currently available for identifying patients with oligometastatic disease, in the clinic. However, imaging modalities fail to detect occult metastasis early, and biomarkers to assess tumor burden dynamics for surveillance during and after treatment are needed.

There are several limitations of this study that need to be noted. First, the retrospective study might result in a potential selection bias. Second, as the limitation of sample size and center number, we failed to conduct external validation. Third, only 118 patients in this study cohort underwent pretreatment plasma EBV DNA detection. Thus, it was hard to estimate the potential prognostic value of EBV DNA. Finally, innovations in chemotherapeutic regimens and advances in radiotherapeutic technology have inevitably changed treatment protocols, although the general treatment principles have remained unchanged.

In conclusion, we established an easy-to-use nomogram which demonstrated good performance in predicting survival and provided individualized stratification for patients with metastatic NPC. Using the prognostic model, patients who are sensitive to local treatment can be identified. In the future, multicenter external validation and prospective studies are needed to generalize the use of our model.
